# Single-cell omics in plant biology: mechanistic insights and applications for crop improvement

**DOI:** 10.1007/s44307-025-00074-8

**Published:** 2025-07-02

**Authors:** Tao Zhu, Tianxiang Li, Peitao Lü, Chenlong Li

**Affiliations:** 1 State Key Laboratory of Biocontrol, Guangdong Provincial Key Laboratory of Plant Stress Biology, School of Life Sciences, Sun Yat-Sen University, Guangzhou, 510275 China; 2 National Key Laboratory for Tropical Crop Breeding, Institute of Tropical Bioscience and Biotechnology &, Sanya Research Institute, Chinese Academy of Tropical Agricultural Sciences, Sanya, 572024 China

**Keywords:** Plant single-cell omics, Single-cell transcriptomics, Biology Findings, Plants, Crops, Horticultural plants

## Abstract

In recent years, single-cell omics technologies have significantly advanced plant and agricultural research, providing transformative insights into plant development, cellular heterogeneity, and environmental response mechanisms. Traditional bulk-level analyses often obscure differences between individual cells, whereas single-cell RNA sequencing (scRNA-seq) and single-nucleus RNA sequencing (snRNA-seq) now reveal unique expression profiles across distinct cell populations, facilitating the identification of novel cell types and elucidation of gene regulatory networks. Additionally, epigenomic approaches like single-nucleus ATAC sequencing (snATAC-seq) offer a deeper understanding of chromatin accessibility and its complex relationship with gene regulation. These technologies have seen widespread application in model plants such as *Arabidopsis thaliana*, as well as in major crops and horticultural plants, providing essential data for crop improvement and breeding strategies. Moving forward, with the continued development and integration of single-cell multi-omics technologies, there will be greater depth of insight into cell-type-specific regulation and complex trait analysis, bringing new opportunities for sustainable agriculture and crop improvement.

## Introduction

Embryophytes are composed of various tissues, organs, and cell types that coordinate with each other to perform specific biological functions (Kane and Higham 2015). Bulk RNA-seq techniques have typically employed plant tissues, organs, or even entire organisms as samples to analyze the molecular mechanisms underlying plant development and responses to environmental signals. However, this method averages the signals from heterogeneous cell populations, masking the individual contributions of distinct cell types and failing to reveal cell-type-specific gene expression profiles (Li and Wang 2021). To address this limitation, single-cell omics technologies have emerged as critical tools for resolving the complex cellular landscapes of plants at the cellular level (Kashima et al. 2020). Compared to traditional RNA-seq, scRNA-seq allows for the clustering and classification of cells within complex tissues or organs, revealing the specific expression profiles and functional characteristics of different cell populations, as well as identifying rare cell subtypes undetectable with bulk RNA-seq (Seyfferth et al. 2021; Shahan et al. 2021; Shaw et al. 2021).

Over the past decade, single-cell omics technologies have been widely applied to study cellular heterogeneity in animals and microorganisms (Marcy et al. 2007; Navin et al. 2011; Wang et al. 2012). With the continuous advancements in sequencing technologies and sample preparation methods, single-cell transcriptome studies have also become more prevalent in plants (Han et al. 2017; Nelms and Walbot 2019; Ryu et al. 2019). Currently, scRNA-seq has been extensively used to define cellular heterogeneity in different plant tissues and organs, construct developmental trajectories, and decode gene regulatory networks (GRNs) (Melnekoff and Lagana 2022; Mo and Jiao 2022; Zheng et al. 2023; Islam et al. 2024). Moreover, single-cell omics technologies have been utilized to investigate plant-environment interactions, providing new insights into how specific tissues, cell types, or individual cells respond to environmental stimuli (Tenorio Berrio and Dubois 2024). In addition, snATAC-seq has been developed to characterize chromatin accessibility across different cell populations at the single-cell level, further offering new dimensions to the study of genome regulation in plants (Farmer et al. 2021; Marand et al. 2021). Notably, tissue preparation for scRNA-seq results in the loss of spatial information of the cells, whereas capturing the spatial positioning of distinct cells is critical for uncovering cell–cell and cell-environment interactions. In contrast, single-cell spatial transcriptomics technologies, by integrating imaging and sequencing, maps the locations of specific transcripts within tissues, elaborates the spatial distribution patterns of gene expression, and thereby enables a more comprehensive molecular-level study of plant tissues (Sang and Kong 2024).

In this review, we aim to summarize the methodologies and applications of single-cell omics technologies in plant science. We begin by introducing the technical principles of single-cell sequencing in plants, including sample preparation, library construction, data analysis, and existing single-cell omics databases. Following this, we reviewed the current progress of single-cell omics studies in model plants such as *Arabidopsis thaliana*, and in crop species including rice, maize, and wheat, as well as horticultural plants. We also discuss the challenges faced by single-cell technologies and how these challenges are likely to shape and accelerate plant-specific research in the future.

## Principles of single-cell transcriptomics in plants

**Fig. 1:**
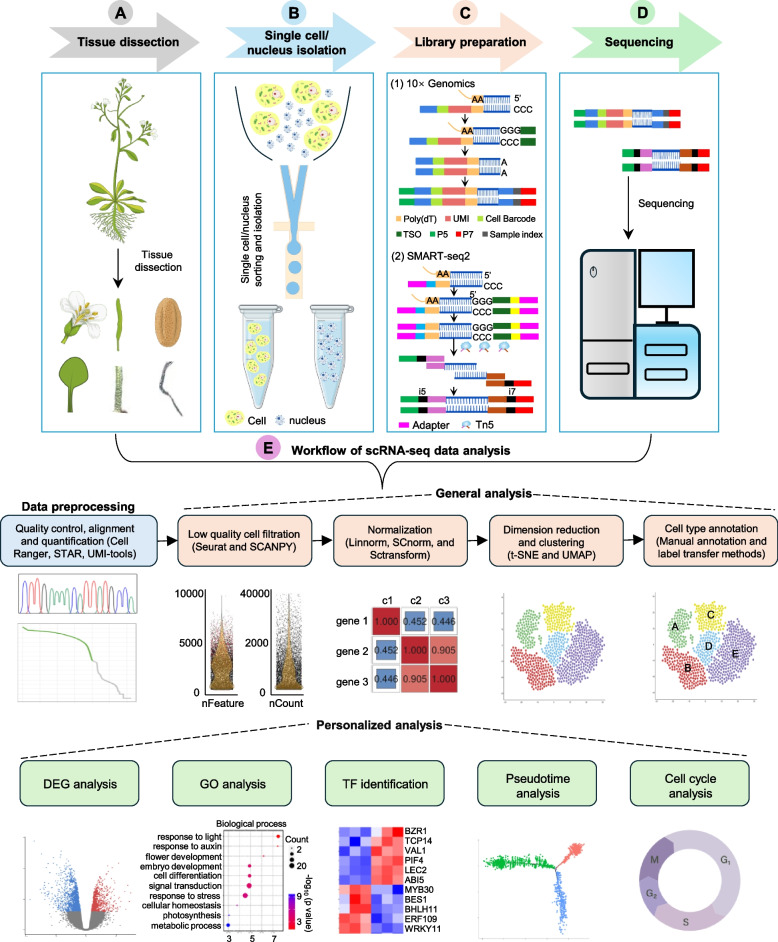
Conceptual workflow of scRNA-seq in plants. The flowchart of a typical scRNA-seq project. **A** Tissue dissection and preparation of single cell/nucleus suspensions. **B** Obtain single-cell/nucleus suspensions through the microfluidic platform. **C** Overview of two major library construction methods for single-cell transcriptomes. (1) The 10 × Genomics and Drop-seq methods utilize beads coated with combinatorial probes to capture transcripts from cell lysates. Subsequently, the tagged reads are pooled and subjected to enzyme- mediated fragmentation, end-repair, and ligation to sequencing adapters. (2) SMART-seq2 captures and amplifies transcripts using a similar mechanism, followed by fragmentation and tagging of the cDNA library with Tn5. **D** Single cell sequencing by selected platform. **E** Workflow of scRNA-seq data analysis. The classic roadmap mainly consists of data preprocessing (blue panel), general analyses (orange panel) and personalized analyses (green panel). Data preprocessing includes quality control, alignment and quantification; general analyses include low-quality cell filtering, normalization, dimension reduction, clustering and annotation of cell types; personalized analyses include DEG analysis, GO analysis, TF idenfication, Pseudotime analysis, and cell cycle analysis. The plot below each box gives a schematic of the visualized results in each analysis step. STAR, Spliced Transcripts Alignment to a Reference. UMI-tools, Unique Molecullar Identifier-tools. SCANPY, Single-Cell Analysis in Python. Linnorm, Linear Model and Normality based Normalization and Transformation Method. DEG, differentially expressed gene. TF, transcription factor

In contrast to animal cells, a defining feature of plant cellular architecture is the presence of a rigid and semi-permeable cell wall. This structural characteristic imposes distinct technical requirements when performing scRNA-seq in plant systems. Specifically, the protocol necessitates enzymatic digestion of plant tissues to degrade the cell wall and yield viable protoplast suspensions. Following successful isolation of single-cell protoplasts, the workflow proceeds with whole-transcriptome amplification and high-throughput sequencing. The resulting datasets are subsequently subjected to comprehensive bioinformatic analyses to resolve cell-type-specific transcriptional profiles (Hwang et al. 2018) (Fig. 1).

### Sample preparation

Sample preparation typically involves the isolation of protoplasts or nuclei from various plant tissues. Isolating protoplasts from plant tissues effectively captures RNA from both the cytoplasm and the nucleus, which facilitates a more comprehensive understanding of gene expression profiles and regulatory networks within individual cells. However, the use of protoplasts in scRNA-seq experiments also has its limitations. One significant drawback is that the enzymatic digestion of the cell wall during protoplast isolation may inadvertently alter the gene expression of certain cells, potentially skewing the accuracy of the results. Additionally, in tissues with particularly robust cell walls, such as xylem, the efficiency of enzymatic digestion may be insufficient, causing detection bias toward cell types that are easier to dissociate (Reed and Bargmann 2021; Cosgrove 2024). Consequently, directly extracting nuclei for scRNA-seq samples has emerged as a viable alternative. This method, which we referred as snRNA-seq thereafter, circumvents the need for cell wall digestion, thereby avoiding gene expression changes induced by enzymatic treatments and proving especially useful for tissues that are difficult to digest (Conde et al. 2021; Farmer et al. 2021).

To ensure optimal outcomes in scRNA-seq experiments, the protoplast or nuclear suspensions must maintain a high degree of integrity with minimal cellular debris. Given that some tissues (e.g., xylem) are challenging to digest enzymatically, fluorescence-activated cell sorting (FACS) has emerged as an effective alternative. This technique not only allows for efficient separation and purification of nuclei but is also applicable for extracting nuclei from frozen tissue, thereby reducing sample preparation time (Wang et al. 2023).

In summary, when selecting a sample preparation method for plant single-cell RNA-seq experiments, researchers must weigh the suitability of protoplasts versus nuclei based on their specific research objectives, the characteristics of the plant species involved, and the breadth and depth of transcriptome coverage required. Careful consideration of these factors is essential to identify the most appropriate preparation method, ultimately leading to the acquisition of high-quality single-cell transcriptome data.

### Construction of scRNA-seq libraries

In scRNA-seq library construction, complementary DNA (cDNA) amplification via reverse transcription of single-stranded RNA primarily relies on two methods: polymerase chain reaction (PCR) and in vitro transcription (IVT) (Kolodziejczyk et al. 2015).

PCR-dependent single-cell platforms, such as Drop-seq, 10 × Genomics, and SMART-seq2 (Picelli et al. 2014; Macosko et al. 2015), are widely used in plant research. In Drop-seq and 10 × Genomics platforms, individual protoplasts or nuclei were captured within single droplets, which contain enzymes and other chemical components for cell lysis and cDNA synthesis, as well as beads coated with DNA oligonucleotide probes comprising droplet-specific barcodes, transcript-specific barcodes (Unique Molecular Identifiers, UMIs), and poly-T sequences. Transcripts released from captured individual protoplasts or nuclei anneal to these probes, while reverse transcription, template switching, fragmentation, and end repair occur sequentially at the 3’ end of the transcripts. Droplets are pooled together after cDNA synthesis for subsequent cDNA collection, purification, and amplification to prepare the sequencing library. In contrast, SMART-seq2 employs a reverse transcription strategy similar to those of Drop-seq and 10 × Genomics but utilizes Tn5 transposase to simultaneously fragment and label cDNA, enabling subsequent library amplification (Fig. 1).

IVT-dependent single-cell platform, such as CEL-seq2, has also been adapted for plant research. In CEL-seq2, reverse transcription is initiated within individual cell-containing wells by primers bearing a UMI, a cell barcode, and a T7 promoter sequence, yielding first-strand cDNA. Following second-strand synthesis to produce double-stranded cDNA, all reactions are pooled and subjected to in vitro transcription via the T7 promoter to amplify RNA. The amplified RNA is then fragmented to an optimal length, 3’-end ligated to sequencing adapters, and reverse-transcribed back into cDNAs, which are PCR-amplified to construct the final sequencing library.

### ScRNA-seq data analysis

The data processing workflow of plant single-cell transcriptomics is generally divided into three steps: the generation of an expression matrix from raw sequencing data; the classification of cell types based on the expression matrix; in-depth annotation and personalized analysis based on the matrix and additional data resources (Gurazada et al. 2021; Denyer and Timmermans 2022; Marand and Schmitz 2022). Next, we will summarize the key tools currently used in single-cell data analysis.

First, for raw data obtained through the 10 × Genomics platform, Cell Ranger serves as an ideal tool for generating the expression matrix (Zheng et al. 2017). Additionally, STAR, UMI-tools, and Salmon are also ideal alternatives (Dobin et al. 2013; Smith et al. 2017; Srivastava et al. 2019). This initial filtering removes cells with transcript counts similar to background levels or cells that are damaged (Fraction Reads in Cells < 85%). During this step, researchers must assess whether a sufficient number of valid cells have been captured, as inadequate capture could lead to incomplete coverage of cell types (Chen et al. 2021b). Notably, the number of captured cells in published studies predominantly ranges from 5,000 to 10,000.

Second, deeper processing of the filtered data is required, which involves steps such as filtering, standardization, normalization, screening of highly variable genes, principal component analysis (PCA), clustering, and cell type annotation (Chen et al. 2021b). In single-cell data filtering workflows, Seurat and SCANPY stand as two widely adopted analytical tools (Wolf et al. 2018; Hafemeister and Satija 2019). Seurat are R-based, which enables them to leverage R-only packages more seamlessly. In contrast, SCANPY is based on Python, allowing for better integration with Python-only packages and deep learning models. At this stage, cells are typically filtered based on their detected gene counts, with most studies requiring a median of over 700 genes per cell. When dealing with plant single-cell data, special attention should be given to chloroplast-related genes. In particular, when the primary research emphasis lies on the regulatory mechanisms governing gene expression within plant nucleus, the elimination of chloroplast genes can effectively mitigate the interference stemming from extraneous information. Moreover, genes whose expression is known to be affected by enzymatic digestion during protoplast isolation also require to be removed (Denyer and Timmermans 2022).

Following filtering, standardization and normalization are carried out using tools such as Linnorm (Yip et al. 2017), SCnorm (Bacher et al. 2017), or sctransform (Hafemeister and Satija 2019), which help in better detecting low-expression genes without their signals being overwhelmed by highly expressed genes. Evaluation based on diverse single-cell datasets and the silhouette width metric showed that the performance of these three methods was comparable (Chen et al. 2021b). After highly variable genes are identified, clustering methods like Partition-based Graph Abstraction (PAGA) can be applied to group cells with similar characteristics (Wolf et al. 2019). These cell groups can then be visualized in a two-dimensional coordinate system using techniques like t-distributed stochastic neighbor embedding (t-SNE) or uniform manifold approximation and projection (UMAP) (Laurens and Hinton 2008; Becht et al. 2018). Lastly, differentially expressed genes (DEGs) for each cell cluster are determined using methods such as the *t*-test (Mishra et al. 2019) or the Wilcoxon rank-sum test (Liang et al. 2021), and the correspondence between DEGs and known marker genes forms the basis for annotating cell groups. Notably, single-cell subpopulation annotation methods are divided into two categories: manual annotation and label transfer. Manual annotation relies on cell type-specific marker genes documented in literature or databases, verifying the expression of these markers within subpopulations using visualization tools, and manually determining cell types based on expert knowledge. Label transfer methods, in contrast, leverage pre-annotated reference datasets. This approach aligns query data with reference data through computational algorithms to automatically assign cell labels based on gene expression similarity. For instance, Single-cell Recognition of cell types (SingleR) performs annotation by calculating expression correlation between query cells and reference cell types (Aran et al. 2019). Multi-task single cell assignment (MtSC) integrates multiple reference datasets (even cross-species) and improves annotation accuracy via deep metric learning, proving high efficacy in heterogeneous data scenarios (Duan et al. 2021). Finally, more selective analyses are performed depending on the research objective, such as trajectory inference and gene regulatory network (GRN) inference (Tripathi and Wilkins 2021; Zheng et al. 2021). This stage of analysis is highly personalized, requiring flexible adjustments and extensions based on the central research questions.

### Plant ScRNA-seq databases

With the rapid accumulation of scRNA-seq data for various plant species, there is an urgent need to establish online databases for public sharing and utilization of these scRNA-seq resources.

The Plant Single Cell Hub (PsctH), PlantscRNAdb, and Plant Cell Marker Data Base (PCMDB) are three comprehensive databases specialized in plant single-cell RNA analysis (Chen et al. 2021a; Xu et al. 2021; Jin et al. 2022). These repositories curate marker genes for diverse cell types in both model and non-model plant species, while providing access to original research materials, associated expression matrices, and other relevant resources. PsctH additionally features a plant marker gene database with all marker genes experimentally validated through RNA in situ hybridization or GFP reporter assays. This database also offers a practical pipeline for protoplast preparation, establishes a standardized workflow for plant scRNA-seq data analysis, and provides R scripts to facilitate data mining. In contrast, PlantscRNAdb includes an online BLAST tool to search for homologous cell-type marker genes across species. PCMDB, on the other hand, integrates two online tools-SingleR and SCSA-for cell type annotation based on marker genes.

The Root Cell Atlas in Rice (RCAR) focuses on root tip tissues of two rice cultivars (japonica NIP and indica 93–11), serving as a comprehensive platform for single-cell transcriptomic data that supports queries for specific cell types, gene identifiers, genome browsing, and visualization. This resource enhances the utility and deep mining of rice single-cell data (Liu et al., 2021b).

Another repository, scPlantDB, compiles 67 high-quality single-cell datasets from 17 plant species, which underwent rigorous manual curation, quality control, and standardization (He et al. 2024). It enables flexible comparative analyses across species, datasets, cell types, and markers. Such comprehensive exploration of plant single-cell data is critical for understanding cellular diversity and evolutionary processes.

## Application of single-cell sequencing in *Arabidopsis*

### Cellular heterogeneity and regulatory networks in plant tissue development

**Fig. 2:**
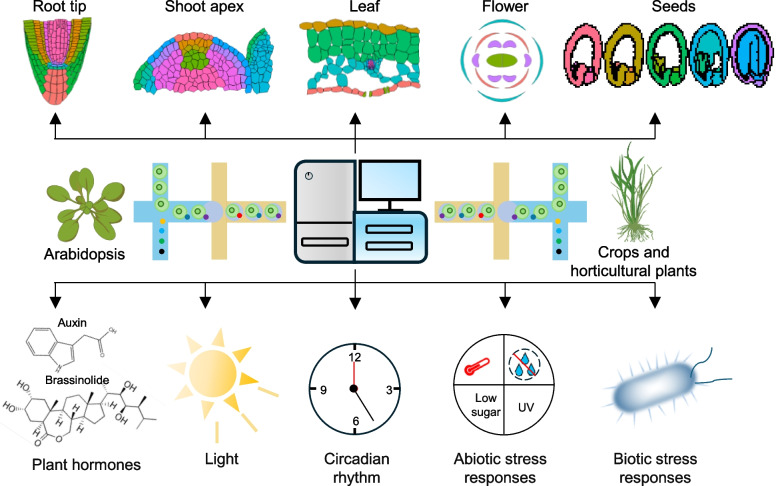
The utilization of single-cell sequencing technology in plants. Single-cell sequencing technology has enabled systematic characterization of transcriptomic profiles at cellular resolution in *Arabidopsis*, crop, and horticultural plants, uncovering cellular heterogeneity and differentiation trajectories within different tissues during developmental processes. Furthermore, this approach has delineated cell type-specific regulatory mechanisms underlying plant responses to phytohormonal cues, environmental signals, and biotic/abiotic stresses. These high-resolution molecular maps provide critical insights into the spatiotemporal coordination of adaptive mechanisms in plant systems

Accurately identifying various cell types in plant tissues or organs is critical for understanding the cell-type-specific functions during tissue development. In the following sections, we explore the application of single-cell transcriptomics technology in diverse plant tissues, highlighting its impact on our understanding of developmental processes (Fig. 2).

#### Cellular heterogeneity in root apical development

The root tip has emerged as the most tractable model system in plant single-cell sequencing research, owing to its highly organized cellular architecture, well-defined differentiation trajectories, and the availability of cell type-specific molecular markers. To date, single-cell transcriptomics technology has enabled the construction of comprehensive cellular atlases spanning from the root apex to mature zones, systematically characterizing molecular signatures across three functional domains: (1) the meristematic zone (encompassing the quiescent center, stem cells, and their progeny), (2) the ground tissue system (epidermis, cortex, and endodermis), and (3) the vascular system (pericycle, xylem, and phloem) (Denyer et al. 2019; Jean-Baptiste et al. 2019; Ryu et al. 2019; Shulse et al. 2019; Zhang et al. 2019; Shahan et al. 2022). Notably, this approach overcomes limitations of traditional cell sorting by identifying rare cell populations, such as QC cells within the stem cell niche, and capturing dynamic transcriptional states of transitional cells during lateral root primordia formation (Denyer et al. 2019; Zhang et al. 2019). These breakthroughs have laid a critical foundation for unraveling the spatiotemporal regulatory networks governing root development.

At the mechanistic level, integration of scRNA-seq data from wild-type and *fer-4* mutant roots revealed that loss of FERONIA (FER) kinase induces cell-type-specific transcriptional reprogramming in root hair differentiation zones (Xu et al. 2024). Subsequent molecular dissection demonstrated that FER stabilizes the transcription factor PHYTOCHROME-INTERACTING FACTOR 3 (PIF3) via phosphorylation, thereby regulating mechanoresponsive target genes in root cap cells (Xu et al. 2024). This regulatory axis offers novel insights into how roots overcome soil mechanical impedance during growth. Equally transformative are multi-omics integration strategies for decoding cell fate determination. Combined snATAC-seq and snRNA-seq analyses have revealed that chromatin accessibility dynamics at regulatory elements precede transcriptional activation during the transition from root tip initial cells to differentiated states (Farmer et al. 2021; Liu et al. 2024). This “pre-programmed” chromatin state suggests an epigenetic priming mechanism underlying cellular specialization, providing a conceptual framework for understanding root stem cell niche homeostasis. Notably, while the study by Xu et al. (2024) revealed the transcriptional regulation of the FER-PIF3 pathway in mediating the mechanosensory response of root cap cells, it did not explain why this pathway is specifically activated in root cap cells rather than other cell types. In contrast, multi-omics studies by Farmer et al. (2021) and Liu et al. (2024) offered a plausible explanation from the perspective of epigenetic regulation: the promoter regions of PIF3 target genes (e.g., mechanosensitive genes) in root cap cells exhibit a pre-existing open chromatin state, allowing PIF3, once activated by FER kinase, to directly bind these regulatory elements and rapidly initiate gene expression in root cap cells.

In addition, current single-cell transcriptomic datasets predominantly focus on cell type annotation, while systematic dissection of gene co-expression networks remains underexplored. Recent studies employing SingleCellGGM algorithm have identified 149 spatiotemporally restricted gene co-expression modules (GEPs) from *Arabidopsis* root scRNA-seq data (Han et al. 2024). These GEPs not only map precisely to developmental trajectories of specific cell types but also uncover cell-type-specific metabolic regulatory networks. Moreover, GEPs encompass a wealth of candidate genes, offering valuable resources and insights for further in-depth investigation of the associated biological processes.

#### Cellular heterogeneity in shoot apex and leaves development

The shoot apical meristem (SAM) and leaves, as core organs governing the development and function of plant aerial parts, have witnessed the evolution of their single-cell research from cell type identification to dissection of spatiotemporal dynamic mechanisms. This progression provides a critical lens for understanding the morphogenesis and functional adaptation of plant above-ground structures. The shoot SAM in plants maintains a continuous production of aerial structures throughout the plant’s lifecycle, serving as a critical hub of cellular and developmental activity. ScRNA-seq studies have revealed that the *Arabidopsis* shoot apex comprises at least 23 transcriptionally distinct clusters classified into seven major cell groups, including mesophyll, guard, shoot meristem, companion, epidermal, cortical, proliferating, and vascular cells (Zhang et al. 2021a). Compared with earlier studies based on bulk RNA sequencing at the tissue level, this work advances the resolution of the SAM from the tissue scale to the level of cellular subpopulations. These findings provide a critical framework for elucidating the molecular mechanisms underlying the continuous generation of aerial structures by the SAM.

In leaf development, scRNA-seq has provided significant insights into transcriptional regulation and cellular differentiation. Liu et al. delineated 10 transcriptionally distinct cell clusters and identified novel leaf vein-specific marker genes of 3-day *Arabidopsis* cotyledons (Liu et al. 2022b). Further analysis demonstrated that CYCLING DOF FACTOR 5 (CDF5) and REPRESSOR OF GA (RGA) may function as central regulatory hubs orchestrating cell fate specification in vascular precursor cells (Liu et al. 2022b). Kim et al. further established the single-cell transcriptome atlas of the leaf vascular system by enriching vascular cells from mature leaves, uncovering the functional module characteristics of the mature vascular system (Kim et al. 2021). These findings provide a comprehensive characterization of the cellular heterogeneity within the leaf vascular system, offering a high-resolution cellular atlas that supports a deeper understanding of the functional roles and dynamic regulatory mechanisms of vascular tissues throughout leaf development. ScRNA-seq analyses of 5-day *Arabidopsis* cotyledons resolved 11 distinct cellular clusters functionally associated with stomatal ontogeny. This finding delineated transcriptional networks driving the transition from meristemoid mother cells to guard mother cells, emphasizing the intricate regulation of stomatal lineage progression (Liu et al. 2020). Moreover, scRNA-seq of *Arabidopsis* leaf epidermal cells identified two bZIP-domain transcription factors, bZIP25 and bZIP53, which are highly expressed in pavement and early protodermal cells, playing key roles in epidermal cell differentiation. These findings, together with previous studies on leaf vascular tissues development, have refined our understanding of overall leaf development at different cellular levels. In addition, spatial enhanced resolution omics sequencing (Stereo-seq) technology uncovered subtle yet significant transcriptional differences between the upper and lower epidermal cells of *Arabidopsis* leaves, identifying a cell type-specific gene expression gradient from the midrib to the leaf margin and revealing distinct spatial developmental trajectories of vascular and guard cells (Xia et al. 2022). In summary, these studies have systematically and extensively elucidated the mechanisms of transcriptional regulation and cell differentiation during leaf development, integrating multiple dimensions such as developmental stages, cellular heterogeneity, and spatial organization.

Notably, the latest single-cell research has obtained over one million high-quality single-nucleus transcriptomes from 20 tissue samples covering the key stages of the entire life cycle of various tissues in *Arabidopsis*. The study innovatively proposed the concepts of SAG-index and YAG-index, which has transformed the description of leaf senescence degree from traditional qualitative methods to quantifiable values, and depicted the senescence trajectory of leaves at the single-cell level (Guo et al. 2025). Meanwhile, the research associated leaf senescence with the nutrient flow in the whole plant, mapped the distribution pattern of carbon and nitrogen elements transported from senescent leaves to sink organs such as roots, flowers, and siliques (Guo et al. 2025). Thus, this study not only advances our understanding of nutrient allocation strategies in plants but also provides a crucial theoretical framework for improving nutrient use efficiency in crops and promoting the development of sustainable agriculture.

#### Cellular heterogeneity in reproductive organs development

In angiosperms, female gametophytes originate from somatic cells and eventually differentiate into the embryo sac within the ovule, a specialized reproductive organ. ScRNA-seq technology has been instrumental in elucidating the developmental trajectories and molecular mechanisms underlying this process. Analysis of *Arabidopsis* ovule primordia at three distinct stages of female gametophyte differentiation identified the major cell types within the female gametophyte and surrounding nucellus, along with their characteristic gene expression profiles. These findings highlighted key regulatory pathways involved in cell fate determination during ovule development (Hou et al. 2021). Moreover, scRNA-seq in germinating *Arabidopsis* seeds systematically delineates the cell type-specific dynamic patterns of gene expression and their associations with functional transitions during germination, underscoring the complexity and coordination required for proper seedling establishment (Liew et al. 2024). These two studies not only validated the universality of scRNA-seq technology in studying the full cycle of plant reproductive development but also, through systematic data integration, laid the foundation for constructing a comprehensive molecular regulatory atlas of the process from germ cell differentiation (female gametophyte formation) to the initiation of postembryonic development (seed germination).

#### Cellular heterogeneity in callus development

Callus holds an important position in modern genetic research. It is a key material for gene function research and plant genetic transformation, as well as an effective way for germplasm resource innovation and preservation. Callus tissue is formed through dedifferentiation of young hypocotyl or shoot apical tissues during plant tissue culture, a process that endows callus with a strong proliferative capacity. The application of single-cell sequencing technology in the study of plant callus regeneration and differentiation reveals the diversity of callus cells and the dynamic changes in cell fate during the regeneration process. scRNA-seq analysis of *Arabidopsis* hypocotyl-derived callus, following six days of induction in a suitable medium, revealed that callus tissue structurally resembles root primordia or the root apical meristem. The central cell layer within the callus displayed transcriptional characteristics of quiescent center cells, which are crucial for the callus’s organ regeneration capacity (Yin et al. 2024). Additionally, through profiling gene expression and identifying cellular subpopulations in wounded regions during root formation, scRNA-seq analyses have revealed several key regulators of leaf adventitious root regeneration (Liu et al. 2022a). Thus, these studies not only elucidate the molecular mechanisms underlying cell fate transition during callus induction, but also provide a theoretical foundation for enhancing callus induction efficiency and optimizing plant regeneration systems.

### Unraveling cell-type-specific regulatory networks underlying plant environmental signal responses

Recent advancements in scRNA-seq technology have enabled a deeper exploration of cellular heterogeneity in plant responses to internal (such as auxins and brassinosteroids) or external signals (such as light) at the single-cell level (Fig. 2). These insights have significantly advanced our comprehensive understanding of the regulatory mechanisms governing plant responses to signals.

#### Plant hormone responses

The precise regulation of the cell cycle is essential for the coordinated development and differentiation of multicellular organisms. In plants, endoreplication is frequently observed in terminally differentiated cells, yet the role of phytohormone in mediating the transition from the mitotic cell cycle to the endocycle, as well as its mechanisms in sustaining the endocycle state, remains unclear. scRNA-seq analyses of root tip tissues have provided critical insights into this process. The results demonstrated that, during endocycle progression, genes involved in auxin synthesis, influx, and efflux are upregulated at specific cell stages, whereas such induction is absent during mitotic cell cycling, suggesting that auxin fluctuations contribute to the maintenance of the endocycle state (Torii et al. 2020).

In addition, scRNA-seq analysis of stomatal lineage cells activated by brassinosteroid (BR) signaling has revealed how the scaffold proteins POLAR and PL1 specifically protect BIN2 (a negative regulator of the BR signaling pathway) from BR signal inactivation in stomatal precursor cells, thereby regulating stomatal development (Kim et al. 2023). These findings highlight the cell-type-specific interpretation of hormonal signals and their role in initiating distinct cellular responses that ensure correct cellular patterning (Kim et al. 2023). Furthermore, a time series scRNA-seq study of the BR-responsive gene regulatory network in the *Arabidopsis* root has defined the elongating root cortex as the primary site of BR-regulated gene expression (Nolan et al. 2023). By reconstructing the developmental trajectory of cortex cells, the study demonstrated that BR signaling specifically promotes the expression of cell wall-related genes, including *HOMEOBOX FROM ARABIDOPSIS THALIANA 7 (HAT7)* and *GT-2-LIKE 1 (GTL1)*, in elongating cortex cells, thereby facilitating the transition from proliferation to elongation (Nolan et al. 2023). Therefore, by mapping the brassinosteroid-mediated spatiotemporal gene regulatory network, this study further elucidates the molecular mechanisms through which plant hormones dynamically govern cell fate determination and developmental progression.

#### Plant light signal and circadian clock responses

Light serves as both an energy source and a key regulatory signal in plant development. Seedling de-etiolation exemplifies light-regulated development, characterized by extensive transcriptomic reprogramming (Jiao et al. 2007). scRNA-seq analyses of *Arabidopsis* seedlings under etiolated, de-etiolating, and light-grown conditions identified 48 distinct cell clusters in stem and root tissues (Han et al. 2023). Developmental trajectory mapping highlighted light’s role in regulating stomatal guard cell specialization and vascular bundle development (Han et al. 2023). Comparative analyses of wild-type and *pifq* mutants revealed that phytochrome-interacting factors (PIFs) regulate the expression of target genes in a cell-type-specific manner, contributing to the differential development of endodermal and stomatal lineage cells (Han et al. 2023). Therefore, this study not only elucidated how light signaling regulates the de-etiolation process and cell fate determination in a cell type-specific manner but also elucidated the specific roles of PIF transcription factors across distinct cell types and their cell type-specific regulatory mechanisms, thereby providing novel insights into how plants modulate cell fate determination through light signaling. Moreover, snRNA-seq profiling revealed that light-induced PTS introns were predominantly observed in mesophyll cells during seedling de-etiolation (Yan et al. 2024). Further analysis demonstrated that the splicing-related factor PROTEIN ARGININE METHYLTRANSFERASE 5 (PRMT5) acts in concert with the E3 ubiquitin ligase CONSTITUTIVE PHOTOMORPHOGENIC 1 (COP1) to coordinately regulate light-responsive PTS events in mesophyll cells (Yan et al. 2024). This study demonstrates that post-transcriptional splicing of cell-type-specific light-responsive genes plays a crucial regulatory role in photomorphogenesis.

Furthermore, scRNA-seq technology has shed light on the molecular mechanisms through which circadian rhythms regulate cell differentiation. The reconstructed actual time series of the differentiation processes at single-cell resolution has revealed that the expression profiles of genes related to the circadian rhythm change before cell differentiation. Among these, the induction of the core clock gene, *LUX ARRYTHMO* (*LUX*), is particularly notable. *LUX* directly targets genes that regulate the cell-cycle progression, thus regulating cell differentiation (Torii et al. 2022). The pre-expression patterns of circadian clock genes prior to cell differentiation provide a mechanistic explanation for the circadian rhythmicity observed during cell fate determination. This finding offers a novel perspective for understanding how circadian rhythms influence plant developmental processes and contribute to the mechanisms by which plants adapt to environmental fluctuations.

### Unraveling cell-type-specific regulatory networks underlying abiotic and biotic stress responses

#### Abiotic stress responses

Abiotic stresses, such as high temperature, drought, ultraviolet (UV) or nutrient deficiency, affect broad regions of plants. However, whether plants exhibit cell-type heterogeneity in response to these stresses was largely unknown. Recent application of scRNA-seq technology has revealed cell-type specificity of plant responses to abiotic stress (Fig. 2).

Single-cell transcriptional profiling revealed that heat stress predominantly activates heat shock transcription factor (HSF) family genes in the outermost root cells including trichoblasts, atrichoblasts, and cortical cells, which are most directly exposed to environmental stress (Jean-Baptiste et al. 2019). This indicates that plant heat responses show significant cell-type specificity, far exceeding the shared responses between tissues.

Under drought stress, WRKY DNA-BINDING PROTEIN (WRKY) transcription factors exhibit notable tissue-specific expression. For instance, WRKY41, WRKY75, and WRKY53 are induced specifically in vascular cells during drought and rehydration, while WRKY8 is predominantly active in trichomes, playing distinct roles in regulating drought responses (Sun et al. 2022b). Furthermore, PIFs mediate the adaptation of leaf epidermis to drought and salt stresses by influencing the development of pavement and guard cells, while root hair cells exhibit heightened sensitivity to osmotic stress (Liu et al. 2022c; Liu et al. 2024).

Nutrient availability also drives cell-type-specific responses. In roots, sucrose deficiency does not alter cell subpopulation composition but significantly impacts the proportions of specific cell types. Differential gene expression under nutrient deprivation shows strong cell-type specificity, with nearly half of the differentially expressed genes restricted to particular tissues or cell types, while only a small fraction is shared across the root (Shulse et al. 2019).

Furthermore, the establishment of a high-resolution transcriptional atlas of *Arabidopsis* palisade mesophyll cells revealed that phenylpropanoid biosynthetic pathway genes exhibit both palisade-specific enrichment and light-responsive regulation (Procko et al. 2022). Mechanistic validation demonstrated that activation of this pathway in palisade cells is essential for biosynthesis of the UV-B-protective metabolite sinapoylmalate. This UV-absorbing compound likely safeguards palisade tissue and adjacent cell layers through high-energy photon attenuation, thereby mitigating UV-induced photodamage to photosynthetic apparatus and genomic integrity (Procko et al. 2022).

In conclusion, single-cell analyses have demonstrated that plant resistance mechanisms exhibit pronounced cell type specificity. Distinct cellular populations differentially respond to environmental challenges by activating specialized transcription factor families or engaging targeted metabolic pathways like the phenylpropanoid biosynthesis route. This mechanistic insight not only deepens our understanding of the regulatory networks governing plant adaptation but also furnishes theoretical foundations for precision breeding.

#### Biotic stress responses

Pathogen infection in host plants is a dynamic and complex process, with different plant cells and tissues exhibiting distinct responses during infection. The application of single-cell sequencing technology in plant pathology, especially in exploring the cellular heterogeneity during plant pathogen invasion and the mechanisms of plant response to viral infections, has made significant progress. These studies also provide new perspectives for developing more targeted plant disease management strategies (Fig. 2).

In the context of *Colletotrichum higginsianum* infection, single-cell transcriptomics revealed that immune receptor genes, particularly those encoding nucleotide-binding leucine-rich repeat receptors (NLRs), were specifically enriched in vascular bundle cells (Tang et al. 2023). Meanwhile, the activation of the abscisic acid (ABA) signaling pathway was found to induce stomatal closure in guard cells at infection sites, effectively limiting pathogen entry through stomatal pores (Tang et al. 2023). Studies using scRNA-seq on *Arabidopsis* leaf tissues during infection by the bacterial pathogen *Pseudomonas syringae* revealed three distinct cellular states that reflect diverse immune strategies (Delannoy et al. 2023). Immune cell populations predominantly express immune-related genes, especially those involved in salicylic acid-mediated defense. Conversely, susceptible cells enriched genes associated with water transport, jasmonic acid, and ABA signaling. Transitional cells displayed intermediate gene expression profiles, with reduced levels of both immune and susceptibility markers (Delannoy et al. 2023). This stratification into immune, transitional, and susceptible states underscores the complexity and dynamic nature of plant immune responses, offering a nuanced understanding of how plants balance defense and susceptibility at the cellular level.

Overall, these studies systematically reveal two core characteristics of plant immune responses. First, different cell types perform differential immune functions through specific immune factors. Second, during the dynamic process from the initial invasion of pathogens to the host’s immune response, cell states show continuous transitions. Therefore, these findings provide precise molecular and cellular targets for innovative disease-management strategies.

## Application of single-cell omics technologies in crops

Recent years, sc/snRNA-seq and snATAC-seq technologies have advanced our understanding of cellular-level regulatory mechanisms that underlie growth, development, and environmental responses in major food crops (Fig. 2).

### Unraveling cellular heterogeneity and regulatory networks in rice growth and development

Liu et al. constructed single-cell transcriptional atlases of the root tips of two rice cultivars, the Japonica group cultivar Nipponbare (Nip) and the Indica group cultivar 93–11, identified the major cell types in rice root tips, and determined novel cell-type-specific marker genes for these two cultivars (Liu et al., 2021b). In addition, by integrating published *Arabidopsis* root scRNA-seq datasets with those from rice, the study revealed conserved and divergent root developmental pathways between dicots and monocots and further identified core conserved genes and species-specific genes in root hair, phloem, and xylem cells (Zhang et al. 2021b). Finally, single-cell transcriptomics provided a comprehensive dynamic atlas of rice inflorescence development, tracing the transition from inflorescence to florets (Zong et al. 2022). This work identified the WOX-family transcription factor DWARF TILLER1 as a key regulator of floral meristem activity and demonstrated the role of the auxin transporter OsAUX1 in inflorescence branching (Zong et al. 2022).

Furthermore, single-nucleus transcriptome sequencing analysis was conducted on unfertilized rice pistils. This analysis deciphered the lineage-specific molecular markers of ovule-derived cells and carpel-derived cells within the pistil tissue, revealing significant heterogeneity between these two progenitor cell populations in terms of transcriptional regulatory networks (Li et al. 2023). Meanwhile, through comparative transcriptome analysis of gametophyte (1N) and sporophyte (2N) cell nuclei, a pivotal pluripotency-resetting event was uncovered in ovule mother cells preceding the sporophyte-gametophyte generation transition (Li et al. 2023). Additionally, spatial single-cell resolution transcriptomic maps of rice embryos revealed gene expression profiles during seed germination, shedding light on regulatory networks associated with nutrient metabolism and hormone signaling (Yao et al. 2024).

In addition, snATAC-seq was utilized to comprehensively dissect the chromatin accessibility landscape of rice root tip cells. The findings indicated pronounced disparities in chromatin accessibility among the principal cell types within the root tips. Concurrently, a cohort of transcription factors intricately associated with accessible chromatin regions (ACRs) was successfully identified (Feng et al. 2022). Moreover, this investigation shed light on the cell-type-specific responses of chromatin accessibility under the influence of heat stress, with the chromatin states of root cap, root hair, and stele cells notably exhibiting heightened sensitivity to thermal stress (Feng et al. 2022).

### Unraveling cellular heterogeneity and regulatory networks in maize growth and development

In maize reproductive biology, scRNA-seq technology has reconstructed the developmental trajectory of male gamete meiosis, revealing a continuous gradient of gene expression preceding meiotic entry, followed by a two-phase transcriptome reorganization during leptotene, during which 26.7% of transcripts exhibited > twofold abundance changes (Nelms and Walbot 2019). Cell cycle gene profiling demonstrated active proliferation of germinal precursor cells, directly refuting the classical hypothesis of stem cell-mediated meiotic initiation (Nelms and Walbot 2019). Further scRNA-seq analyses of inflorescence meristems and florets identified 16 spatiotemporally distinct gene clusters that drive sexual differentiation through redox homeostasis, programmed cell death, and hormone signaling (Sun et al. 2024). Notably, the pistil-protection gene SILKLESS1 (SK1) antagonizes pistil-suppression pathways, establishing a core regulatory network for pistil fate determination (Sun et al. 2024).

In vegetative organ development, single-cell resolution atlases have unveiled mechanisms underlying cortical tissue elaboration. The SHORT-ROOT (SHR) transcription factor exhibits non-canonical expression at the cortex-stele boundary, demonstrating an eight-cell-layer-spanning mobility that drives cortical expansion through SHR signaling (Ortiz-Ramírez et al. 2021). Analyses of the SAM revealed that apical stem cells maintain genome integrity despite low mitotic activity, with ectopic expression of *KNOTTED1* (*KN1*) accelerating differentiation and promoting sheath base morphogenesis (Satterlee et al. 2020). In leaves, integrated scRNA-seq and in situ hybridization delineated the specialization of bundle sheath cells for nutrient transport (Bezrutczyk et al. 2021), while heat shock transcription factors (HSFs) and CONSTANS-like (COL) factors were identified as master regulators of early and late mesophyll cell transcriptional reprogramming, respectively (Tao et al. 2022).

SnATAC-seq profiling of six maize organs, including root tips and inflorescences, has systematically mapped their epigenomic landscape. Cell type-specific *cis*-regulatory elements (CREs) are enriched for enhancer activity and preferentially localize to unmethylated long terminal repeat retrotransposons. These CREs serve as hotspots for phenotype-associated genetic variation and provide key targets for modern molecular breeding (Marand et al. 2021). In disease resistance research, through weighted gene co-expression network analysis (WGCNA) of single-cell data from maize root tips inoculated with *Fusarium verticillioides* (Fv), 12 Fv-responsive regulatory modules were identified. Subsequently, six cell-type-specific immune regulatory networks were constructed using gene network inference with ensemble of trees (GENIE3) and genome-wide association study (GWAS) methods. This study reveals the immune regulatory networks of major cell types in maize root tips at single-cell resolution, thus laying the foundation for deciphering the molecular mechanisms of maize disease resistance (Cao et al. 2023).

Notably, in maize, genetic variations in *cis*-regulatory regions contribute approximately 40% of phenotypic variations in agronomic traits. However, previous studies failed to decipher the influence of the cellular environment on regulatory variations and their underlying molecular mechanisms. The latest research conducted scATAC-seq analysis on 172 maize inbred lines, revealing approximately 22,000 genetic variations associated with chromatin accessibility and establishing the central role of TCP (teosinte branched1, cycloidea, and proliferating cell factor family) transcription factors in chromatin accessibility and three-dimensional genome organization. By integrating multi-omics data, the study further revealed that transposable elements mediate a regulatory-innovation mechanism during maize domestication and adaptation, thereby providing precise targets for maize improvement (Marand et al. 2025).

### Unraveling cellular heterogeneity and regulatory networks in wheat (*Triticum aestivum*) growth and development

Zhang et al. using snRNA-seq and snATAC-seq, has for the first time analyzed the subgenome-specific expression asymmetry and cell type-specific regulatory networks of hexaploid wheat root cells at a single-cell level. Through co-expression network analysis, the study identified cell type-specific transcription factor regulatory modules, providing valuable insights into the biological functions of these transcription factors in wheat root cells and offering a basis for further research on their precise roles (Zhang et al. 2023a).

### Unraveling cellular heterogeneity and regulatory networks soybean growth and development

Recent advances in single-cell omics have significantly enhanced our understanding of nodule development and nitrogen fixation mechanisms in legumes. In the determinate-nodule model crop soybean, a snRNA-seq atlas of roots and nodules at 14 days post-inoculation identified 17 major cell clusters, including six nodule-specific types (Sun et al. 2023). Integrated spatial transcriptomic analysis further revealed uninfected cells in the central infection zone differentiating into functionally specialized subpopulations during nodule maturation, with a transient infected cell subtype enriched in nodulation-related genes (Liu et al. 2023). Single-cell profiling of mature nodules demonstrated functional heterogeneity among *Bradyrhizobium diazoefficiens*-infected cells, including coexisting nitrogen-fixing active and senescent subpopulations. Co-expression network analysis identified novel candidates such as GmFWL3, a plasma membrane microdomain-associated protein essential for rhizobial infection (Cervantes-Pérez et al. 2024).

In addition, single-nucleus transcriptomic mapping of soybean seeds uncovered molecular heterogeneity exceeding anatomically distinguishable cell types across embryo, endosperm, and seed coat compartments. Developmental trajectory analysis highlighted functional transitions among three endosperm sub-cell types, while co-expression networks elucidated regulatory principles underlying spatial patterning during embryogenesis (Pelletier et al. 2024).

Multi-omics integration has revolutionized systemic exploration of soybean gene regulatory networks. A comprehensive transcriptomic atlas combining bulk RNA-seq data from 314 samples across developmental stages with snRNA-seq and spatial omics data from five organs (roots, nodules, shoot apices, leaves, and stems) delineated organ-specific expression modules and spatiotemporal regulatory dynamics of key processes like nodulation (Fan et al. 2025). Complementarily, spatial chromatin accessibility profiling across ten tissues identified 103 cell types and 303,199 accessible chromatin regions (ACRs), with 40% showing cell-type specificity and enrichment for transcription factor motifs governing symbiotic nitrogen fixation. Embryo-endosperm developmental trajectories further revealed functional transitions in endosperm subpopulations and key regulators of cell fate specification (Zhang et al. 2025).

Single-cell approaches have also decoded soybean responses to biotic stress. Systemic analysis of soybean mosaic virus (SMV) infection identified *GmGSTU23* and *GmGSTU24*—tau-class glutathione S-transferases that were upregulated across all leaf cell types during SMV challenge. Transient overexpression in *Nicotiana benthamiana* confirmed their broad-spectrum antiviral activity, highlighting GSTUs as promising targets for disease-resistant breeding in soybean (Song et al. 2024).

### Unraveling cellular heterogeneity and regulatory networks in peanut (*Arachis hypogaea*) growth and development

Single-cell transcriptomic profiling of peanut leaves has systematically deciphered the heterogeneity of leaf cell populations, identifying eight distinct clusters with unique transcriptional signatures. Pseudotime trajectory reconstruction elucidated the developmental pathways of mesophyll and epidermal cells, revealing that palisade cells may transdifferentiate into spongy cells, while epidermal cell lineages originate prior to primordial formation (Liu et al. 2021a). At the molecular level, functional characterization of fatty acid desaturase FAD2 uncovered its dual regulatory mechanism: FAD2 mutation not only elevates oleic acid content but also suppresses leaf growth by downregulating the expression of the cytokinin biosynthesis gene *LOG* in vascular cells (Du et al. 2023). Integrated analysis of single-cell transcriptomes under light and dark conditions demonstrated that darkness disrupts peanut seedling morphogenesis by inhibiting cell cycle progression and chlorophyllide synthesis, whereas light antagonizes auxin signaling to specifically truncate the developmental trajectory of epidermal cells (Deng et al. 2024).

Further integration of single-nucleus RNA-seq and ATAC-seq data from peanut pods precisely resolved cell subpopulations within pod walls, including meristematic, embryonic, and vascular cells. This analysis highlighted the role of MADS-box genes in parenchyma cell differentiation and gravity-responsive genes in vascular cells, implicating the vascular system as a central regulator of geotropic growth in peg tissues (Cui et al. 2024). Additionally, single-nucleus transcriptomic mapping of peanut stems delineated five cell subtypes and their differentiation trajectories. A co-expression network comprising 3,306 core differentially expressed genes revealed the enrichment of auxin signaling pathway components. Notably, tissue-specific overexpression of *AhWRKY70* in stem cortex and xylem cells underscores its potential role in coordinating hormone signaling to modulate stem growth (Wang et al. 2025).

In summary, these studies have constructed single-cell transcriptomic atlases and chromatin accessibility profiles, elucidating cell type-specific characteristics, developmental mechanisms, and regulatory networks in crops. Cross-species comparative analyses further uncovered conserved and divergent developmental trajectories. These advancements lay a robust foundation for deciphering crop growth, reproduction, and environmental stress adaptation, while providing theoretical frameworks and actionable targets for genetic improvement and precision breeding programs.

## Application of single-cell omics technologies in horticultural plants

In recent years, single-cell sequencing technology has been applied in the research on the growth and development of horticultural plants, not only deepening our understanding of plant cellular heterogeneity but also providing new strategies for improving horticultural plant breeding. In the field of horticultural plants, due to the limitations of sample collection (difficulties in single-cell separation due to the rich content of secondary metabolites), single-cell sequencing technology has only been reported in tissues of longan (*Dimocarpus longan*) (Chen et al. 2021b) tomato (*Solanum lycopersicum*) (Omary et al. 2022; Yue et al. 2024), strawberry (*Fragaria vesca*) (Bai et al. 2022), rapeseed (*Brassica napus*) (Li et al. 2024), Chinese cabbage (*Brassica rapa*) (Guo et al. 2022; Sun et al. 2022b), lychee (*Litchi chinensis*) (Yang et al. 2023), and Catharanthus roseus (Sun et al. 2022a) (Fig. 2).

### Unraveling cellular heterogeneity and regulatory networks in horticultural plants development

Studies using Stereo-seq, spatial transcriptomics, and single nucleus RNA-seq on de novo shoot organogenesis (DNSO) in tomato callus have shown that tomato callus cells are heterogeneous. The study identified cell types such as epidermal, vascular tissue, shoot primordia, internal callus, and emerging shoots, indicating significant differentiation of callus cells during regeneration. Further analysis showed that light-induced mesophyll cells play an important role in shoot regeneration of callus, emphasizing the importance of light in plant regeneration (Song et al. 2023). This discovery not only deepens our understanding of the regeneration mechanism of callus but also provides new ideas for light regulation of plant regeneration. Plant embryogenic calli (EC) can complete plant regeneration through somatic embryogenesis. Single-cell transcriptome analysis of longan embryogenic callus identified approximately 30,000 cells, which can be divided into 12 cell clusters, including proliferating cell clusters, vascular cell clusters, and epidermal cell clusters. Further pseudotime analysis depicted a continuous trajectory from early somatic cell division to vascular and epidermal cell differentiation. Co-expression analysis showed that some key transcription factors play an important role in somatic embryogenesis. The study revealed a continuous cell differentiation trajectory from early embryonic cell division to vascular and epidermal cell differentiation mediated by transcription factors, providing a new perspective for understanding somatic embryogenesis in woody plants (Zhang et al. 2023b).

Chinese cabbage is a widely cultivated vegetable crop worldwide, and its leaves, as the main edible organ, are rich in nutrients. In-depth study of the development process of Chinese cabbage leaves and their response mechanisms to adversity is of great scientific and economic value for understanding the molecular basis of plant growth and development, and improving crop stress resistance and yield. Drop-seq revealed the single-cell transcriptome map of Chinese cabbage buds and young leaves. Approximately 30,000 cells from buds and young leaves were divided into 19 gene clusters, which were further divided into meristematic cells, mesophyll cells, proliferating cells, epidermal cells, guard cells, and vascular bundle cells using GO (Gene Ontology) and KEGG (Kyoto Encyclopedia of Genes and Genomes) enrichment and marker gene enrichment (Sun et al. 2022b). Moreover, in Chinese cabbage, prolonged heat stress leads to substantial cell-type specificity in transcriptional responses, with only 242 shared among five major leaf cell types (Sun et al. 2022b). The study provided various cell type-specific marker genes for Chinese cabbage research, providing a theoretical basis for promoting the application of single-cell transcriptome sequencing technology in Chinese cabbage (Sun et al. 2022b). Mesophyll cells (MCs), as the main cell population in leaves, are the main site of plant photosynthesis, and the differentiation of mesophyll cells determines the diversity of leaf morphology. Single-cell transcriptome analysis of Chinese cabbage mesophyll cells divided the cells into eight types, including mesophyll cells, epidermal cells, and guard cells, among which the mesophyll cell group was further divided into palisade mesophyll cells (PMCs) and spongy mesophyll cells (SMCs). PMCs were enriched in photosynthesis-related genes, while SMCs were associated with genes responding to external environmental stimuli. This cell type-specific gene expression reveals the mechanism by which plants optimize their photosynthetic efficiency and environmental adaptability through cell differentiation in response to environmental changes. In addition, the differentiation pattern of mesophyll cells may vary at different developmental stages and under different environmental conditions, indicating that plants can dynamically adjust their cellular composition to cope with external changes (Guo et al. 2022).

Floral transition is a key process in the transition of plants from vegetative growth to reproductive growth. Resolving the floral transition process of horticultural plants at the single-cell level is of great significance for the yield of horticultural crops. Single-cell nuclear transcriptome data of dormant buds, vegetative buds, and floral buds during the flowering process of lychee were constructed and analyzed, detecting 40,000 single-cell nuclei and dividing them into 12 cell populations such as mesophyll cells and epidermal cells (Yang et al. 2023). Comparison of cell populations among the three types of buds showed cellular heterogeneity among different bud types, such as floral bud-specific mesophyll cell populations. Further analysis identified key cell populations that determine bud flowering, ultimately proving that key genes *LcFT1* and *LcTFL1-2*, in the form of mRNA, are transported from leaves to buds and induce the formation of floral organs. The study successfully constructed a single-cell transcriptome map of lychee bud flowering, providing a research basis for the regulation of flowering transition in horticultural plants.

The medicinal plant *Catharanthus roseus* can synthesize monoterpene indole alkaloids (MIAs) that are beneficial to human anti-cancer and blood pressure reduction. Therefore, it is of great significance to resolve the MIA synthesis pathway of *Catharanthus roseus* at the single-cell level. A study using *Catharanthus roseus* as a model constructed a high-resolution single-cell expression map of its leaves using single-cell RNA sequencing technology (Sun et al. 2022a). The study found that different steps of the MIA synthesis pathway occur in different cell types, with the initial steps occurring in parenchyma cells related to internal conductive tissue, intermediate steps occurring in epidermal cells, and late steps completed in other specific cell types. This cell type-specific distribution indicates that MIA biosynthesis is a finely regulated process involving metabolic compartmentalization between multiple cells. This discovery not only provides new insights for optimizing the MIA synthesis pathway but also offers potential targets for improving MIA production through metabolic engineering and synthetic biology (Sun et al. 2022a).

### Unraveling cell-type-specific regulatory networks underlying biotic stress responses in horticultural plants

Plant pathogen invasion is a highly dynamic process involving complex intercellular signaling and responses. The use of snRNA-seq technology in the analysis of transcriptional changes at the cellular level in strawberry leaves infected by *Botrytis cinerea* (Bai et al. 2022) revealed the rapid response of the epidermal and mesophyll cells to the infection. The study showed that during the early stages of infection, the epidermal and mesophyll cells quickly shifted from a normal physiological state to a defense response, accompanied by changes in the expression of specific genes, such as the high expression of transcription factors like *WRKY75* in various cell types. These results indicate that different cell types actively participate in the transcriptional regulation process through their unique gene expression patterns to form a complex regulatory network to resist pathogen invasion. snRNA-seq technology was also utilized to map the single-cell transcriptome of healthy and ToCV-infected tomato leaves (Yue et al. 2024). The results showed that after viral infection, the number of epidermal and palisade tissue cells decreased, while the proportion of trichome cells increased. This dynamic change in cell types suggests that plants may enhance their defense capabilities by adjusting the proportion of cell types during viral invasion. In addition, the study identified the F-box protein SKIP2 as a key factor in maintaining chlorophyll levels during viral infection, emphasizing the core function of the transcription factor ERF4 in regulating the expression of *SKIP2*. By regulating the expression of key genes, plants can maintain the green state of leaves before and after viral infection, mitigating the impact of the virus. These studies not only demonstrate the application value of single-cell sequencing technology in understanding cellular heterogeneity and response mechanisms during plant disease resistance but also provide a foundation for identifying key genes in plant resistance mechanisms. By revealing the specific responses of different cell types to pathogen invasion and viral infections, potential targets are provided for future breeding and gene editing strategies.

In summary, single-cell sequencing can elucidate the complex mechanisms underlying the growth and development, stress resistance, and disease resistance of horticultural plants. The aforementioned studies all indicate that the heterogeneity of plant cells plays a significant role in adapting to developmental and environmental changes. Through single-cell analysis of horticultural plants, we can identify new cell types and key regulatory networks, providing a theoretical foundation for the study of horticultural plants and further innovating genetic improvement and breeding methods. However, due to the limitations of horticultural plant materials themselves, the extraction of protoplasts from some organs such as fruits is challenging, thus technological innovation remains the direction we need to strive for and the driving force for our progress.

## Perspective

In recent years, single-cell technologies have emerged as promising tools in plant research; however, they still face technical challenges in sample preparation. The presence of rigid cell walls surrounding plant cells significantly impedes the isolation of intact nuclei or protoplasts, highlighting the necessity for further development of effective cell dissociation methods (Plant Cell Atlas et al., 2021). Addressing this challenge requires the optimization of protoplast isolation conditions, including enzyme treatment duration, temperature, and osmotic potential, to enhance both the yield and viability of protoplasts (Shaw et al. 2021). Compared to scRNA-seq, snRNA-seq offers advantages for analyzing complex plant tissues, particularly when the presence of cell walls poses challenges to protoplast isolation. However, maintaining the integrity of plant nuclei during the isolation process is critical, as their small size and susceptibility to damage necessitate careful handling and optimization of lysis buffer composition and centrifugation parameters (Wang et al. 2022).

For non-model species, a major challenge in scRNA-seq data analysis is the lack of well-characterized, cell-type-specific marker genes (Cuperus 2022). To tackle this issue, the establishment of databases containing cell-type-specific marker genes across different species is essential. For instance, the PCMDB database includes 81,117 cell marker genes associated with 263 cell types from 22 tissues across six plant species (Jin et al. 2022). Furthermore, the incompleteness or inadequate annotation of reference genomes for many plant species complicates the accurate mapping and interpretation of scRNA-seq data.

Additionally, the integration of multi-omics technologies is anticipated to be a cutting-edge frontier. Advances in single-cell epigenomics, proteomics, and metabolomics from animal research necessitate similar breakthroughs in plant studies. Conducting multi-omics analyses under various environmental conditions will validate existing knowledge obtained through scRNA-seq (Katam et al. 2022). For example, the integration of scRNA-seq with scATAC-seq has successfully constructed transcriptional networks in maize (Marand et al. 2021). Recent progress in spatial transcriptomics has also enabled scientists to examine gene expression patterns in specific areas of plants while preserving their spatial context (Giacomello et al. 2017; Giacomello and Lundeberg 2018). Both 10 × Visium and Stereo-seq are critical platforms in spatial transcriptomics research. 10 × Visium utilizes slides with oligonucleotide-coated spots, including a spatial barcode, UMI, and poly-T sequences, to capture mRNA from tissue sections within 55-μm spots. In contrast, Stereo-seq employs a DNA nanoball array spaced at 500-nm intervals (each containing Coordinate IDs, Molecular IDs, and poly-T sequences) to capture mRNA from distinct tissue locations. 10 × Visium provides a more accessible approach for broad spatial gene expression analysis. However, the resolution of 10 × Visium is constrained by the spacing between capture spots and generally lower than that of Stereo-seq, which can achieve single-cell resolution. Therefore, Stereo-seq, due to its high-resolution capability, demonstrates marked advantages in research areas such as developmental biology, where understanding intercellular interactions and microenvironments requires high precision (You et al. 2024). Additionally, Stereo-seq technology integrates spatial resolution mapping with scRNA-seq, enabling researchers to achieve single-cell resolution while preserving spatial information (Xia et al. 2022). Such structural information is crucial for identifying the locations of rare cell types and elucidating developmental trajectories (Liu et al. 2023). Finally, single-cell ribosome profiling has the potential to explore translation heterogeneity within specific cell populations in plants (Wang and Mao 2023). In summary, the development and integration of single-cell multi-omics approaches in plant research are poised to reshape our understanding of plant development and plant-environment interactions, thereby laying the groundwork for applications in plant breeding, sustainable agriculture, and crop adaptability improvement.

## Data Availability

No datasets were generated or analyzed during the current study.
